# Homologous Recombination Deficiency (HRD) in Cutaneous Oncology

**DOI:** 10.3390/ijms241310771

**Published:** 2023-06-28

**Authors:** Favour A. Akinjiyan, Renee Morecroft, Jordan Phillipps, Tolulope Adeyelu, Andrew Elliott, Soo J. Park, Omar H. Butt, Alice Y. Zhou, George Ansstas

**Affiliations:** 1 Division of Medical Oncology, Department of Medicine, Washington University in Saint Louis, St. Louis, MO 63130, USA; 2 CARIS Life Sciences, Irving, TX 75039, USA; 3 Moores Cancer Center, University of California San Diego, La Jolla, CA 92093, USA

**Keywords:** skin cancer, DNA repair, homologous recombination deficiency, PARP inhibitors, synthetic lethality, genomic scars, immunotherapy, melanoma

## Abstract

Skin cancers, including basal cell carcinoma (BCC), cutaneous squamous cell carcinoma (SCC), and melanoma, are the most common malignancies in the United States. Loss of DNA repair pathways in the skin plays a significant role in tumorigenesis. In recent years, targeting DNA repair pathways, particularly homologous recombination deficiency (HRD), has emerged as a potential therapeutic approach in cutaneous malignancies. This review provides an overview of DNA damage and repair pathways, with a focus on HRD, and discusses major advances in targeting these pathways in skin cancers. Poly(ADP-ribose) polymerase (PARP) inhibitors have been developed to exploit HRD in cancer cells. PARP inhibitors disrupt DNA repair mechanisms by inhibiting PARP enzymatic activity, leading to the accumulation of DNA damage and cell death. The concept of synthetic lethality has been demonstrated in HR-deficient cells, such as those with BRCA1/2 mutations, which exhibit increased sensitivity to PARP inhibitors. HRD assessment methods, including genomic scars, RAD51 foci formation, functional assays, and BRCA1/2 mutation analysis, are discussed as tools for identifying patients who may benefit from PARP inhibitor therapy. Furthermore, HRD has been implicated in the response to immunotherapy, and the combination of PARP inhibitors with immunotherapy has shown promising results. The frequency of HRD in melanoma ranges from 18% to 57%, and studies investigating the use of PARP inhibitors as monotherapy in melanoma are limited. Further research is warranted to explore the potential of PARP inhibition in melanoma treatment.

## 1. Introduction

Skin cancers are the most common type of cancer in the United States, and over 100,000 cases are diagnosed each year [1,2]. There are several main groups of cutaneous malignancies; with basal cell carcinoma (BCC) comprising the majority of new diagnoses, followed by cutaneous squamous cell carcinoma (SCC) and melanoma. The natural history and prognosis of skin cancers are variable, but advanced or metastatic disease can cause significant morbidity and mortality [3,4,5]. The skin is continuously exposed to carcinogenic and environmental insults such as sun damage and chemical exposures. As such, the loss of the DNA repair pathways in the skin can play a major role in tumorigenesis. Only a few studies have explored the possibility of targeting DNA repair pathways in cutaneous cancers as an avenue for treatment. This review discusses DNA damage and repair (DDR) pathways, highlighting major advances in targeting DDR pathways—especially homologous recombination deficiency—in cutaneous malignancies.

## 2. DNA Damage and DNA Damage Repair Pathways

The ability to repair DNA damage is critical to maintaining genomic integrity within a cell. The disruption of normal DNA structure or sequence can lead to DNA damage [6]. This can be caused by both endogenous and exogenous factors such as UV radiation, chemical agents, and errors in DNA replication. When DNA damage occurs, it can lead to mutations, chromosomal abnormalities, and even cell death. To maintain the integrity of the DNA molecule, cells have evolved several mechanisms to repair DNA damage. These mechanisms include:

1. Nucleotide excision repair (NER): NER is the primary DNA repair mechanism involved in removing UV-induced DNA damage, such as cyclobutane pyrimidine dimers (CPDs) and 6–4 pyrimidine–pyrimidone photoproducts (6–4 PPs), which are the most common DNA adducts formed in sun-damaged skin [7,8]. NER involves a multi-step process of recognition, incision, and removal of the damaged DNA strand followed by re-synthesis and ligation.
  Xeroderma pigmentosum (XP) is a rare genetic disorder characterized by defects in NER. Individuals with XP are highly susceptible to developing skin cancers, as their impaired NER function leads to the accumulation of UV-induced DNA damage [9].
2. Base excision repair (BER): BER is involved in repairing DNA damage caused by reactive oxygen species (ROS) and other small base lesions. It primarily deals with single-base lesions, including oxidized bases and DNA base modifications. BER involves the recognition and removal of the damaged base, followed by DNA synthesis and ligation [10].
3. Mismatch repair (MMR): MMR corrects errors that occur during DNA replication, such as mismatches and small insertion/deletion loops. MMR proteins recognize and excise the incorrectly paired base(s), and the DNA is then resynthesized and ligated to restore the correct sequence [11]. Germline mutations in MMR-related genes, as seen in Lynch syndrome, can lead to an increased risk of developing certain types of skin cancer, including sebaceous gland carcinoma.
4. Non-homologous end joining (NHEJ): NHEJ is an error-prone DNA repair pathway that primarily repairs double-strand breaks (DSBs) in DNA. NHEJ operates by directly joining the broken ends of DNA strands without requiring extensive sequence homology [12]. It involves the binding of Ku proteins to the broken DNA ends, the recruitment of additional factors, the processing of the ends, and ligation [13]. NHEJ plays a crucial role in repairing DSBs induced by various factors, including ionizing radiation and reactive oxygen species. It is considered an important repair mechanism in non-dividing cells, such as differentiated cells in the skin.
5. Homologous recombination (HR) repair: HR is a high-fidelity repair mechanism involved in repairing double-strand breaks (DSBs) in DNA. It utilizes an undamaged sister chromatid or homologous DNA sequence as a template for accurate repair [14]. HR is crucial for repairing DSBs induced by ionizing radiation and certain chemotherapeutic agents.

## 3. PARP Inhibition

PARP inhibitors are a class of drugs that target poly(ADP-ribose) polymerase (PARP) family enzymes, specifically PARP1 and PARP2 [15]. PARP enzymes play a crucial role in DNA repair processes, particularly in the repair of single-strand breaks (SSBs) through the base excision repair (BER) pathway. PARP inhibitors exert their therapeutic effect by inhibiting the enzymatic activity of PARP and disrupting DNA repair mechanisms.

When DNA sustains SSBs, PARP enzymes are activated and bind to the damaged site. PARP utilizes nicotinamide adenine dinucleotide (NAD+) to synthesize and attach poly(ADP-ribose) (PAR) chains to itself and other acceptor proteins [16]. This PARylation process attracts and recruits various DNA repair factors to initiate the repair of SSBs through the BER pathway [16]. PARP inhibitors bind to the catalytic domain of PARP enzymes, preventing their enzymatic activity and inhibiting the PARylation process. By doing so, PARP inhibitors impede the repair of SSBs, leading to the accumulation of unresolved SSBs and their conversion into double-strand breaks (DSBs) during DNA replication [16].

## 4. Homologous Recombination Deficiency and Associated Pathways

Homologous recombination deficiency (HRD) refers to a cellular state in which the ability of cells to repair double-strand DNA breaks (DSBs) via the homologous recombination (HR) pathway is impaired [12]. HR is a high-fidelity repair mechanism that uses an undamaged sister chromatid as a template to repair DSBs. In HRD cells, the HR pathway is either absent or impaired, leading to the accumulation of genomic alterations and chromosomal abnormalities. HRD is of particular interest in the field of cancer research, as it is associated with increased sensitivity to PARP inhibitors [17].

## 5. Assessment of HRD

The assessment of HRD can help identify patients who may be sensitive to specific therapies, such as PARP inhibitors. Several assays have been developed to measure HRD, including:

1. Genomic scars/signatures: HRD can leave characteristic genomic scars or signatures, which are specific patterns of genetic alterations or rearrangements detected in the cancer genome [18]. These include large-scale state transitions (LST), telomeric allelic imbalance (TAI), and loss-of-heterozygosity (LOH) events. These genomic scars can be detected using various genomic profiling techniques, such as next-generation sequencing (NGS).
2. RAD51 foci formation: RAD51 is a key protein involved in the HR repair pathway. HRD can be assessed by examining the formation and accumulation of RAD51 foci, which represent ongoing HR repair activity in cells [19]. Immunofluorescence microscopy is commonly used to visualize RAD51 foci.
3. Functional assays: Functional assays measure the ability of cells to undergo HR repair by introducing exogenous DNA substrates harboring specific DNA lesions or DSBs. These assays assess the efficiency and accuracy of HR repair and can provide direct evidence of HRD. Commonly used HRD functional assays include:
  - Homologous recombination reporter assay: In this assay, a reporter construct is introduced into cells, which contains a disrupted fluorescent protein gene (e.g., GFP) between two direct repeats [20]. The cells are then treated to induce DSBs or DNA lesions. HR repair restores the intact fluorescent protein gene by using the intact copy located in the direct repeats. The restoration of fluorescence indicates successful HR repair and can be quantified by flow cytometry or fluorescence microscopy. Decreased HR repair efficiency or impaired HR function in HRD cells results in reduced fluorescence signal.
  - DR–GFP assay: The DR-GFP assay (direct repeat–green fluorescent protein) is a variation of the homologous recombination reporter assay [21]. In this assay, cells are stably transfected with a GFP-based reporter construct containing a recognition site for the I-SceI endonuclease. Introduction of the I-SceI endonuclease induces a site-specific DSB in the reporter construct. HR repair using an exogenously provided donor DNA template results in the restoration of a functional GFP gene. The efficiency of HR repair can be assessed by measuring GFP-positive cells using flow cytometry.
  - Sister chromatid exchange (SCE) assay: The SCE assay measures the exchange of genetic material between sister chromatids, which is indicative of HR activity [22]. Cells are exposed to a DNA-damaging agent, such as a cross-linking agent or a DNA intercalating agent, which induces DNA lesions and subsequent repair. During repair, sister chromatids can exchange DNA segments through HR repair. The frequency of SCEs is assessed by staining the chromosomes and examining them under a microscope. A decreased frequency of SCEs may indicate HRD.
4. BRCA1/2 mutation analysis: HRD is strongly associated with mutations in the BRCA1 and BRCA2 genes, which are key players in the HR repair pathway. Testing for BRCA1/2 mutations can provide information on the presence of HRD. This can be done using various methods, including DNA sequencing, gene panel testing, or specific BRCA1/2 mutation detection assays.

These functional assays provide quantitative and qualitative measurements of HR repair activity in cells and can help identify HRD.

## 6. PARP Inhibitors: Synthetic Lethality in HR-Deficient Cells

PARP inhibitors exhibit synthetic lethality in cells with homologous recombination deficiency (HRD) [23]. In normal cells with functional homologous recombination (HR) repair, DSBs are primarily repaired through HR using an undamaged sister chromatid or homologous DNA as a template. However, in HR-deficient cells, such as those with BRCA1 or BRCA2 mutations, HR repair is compromised. In these cells, the inhibition of PARP further impairs the alternative repair pathway, leading to the accumulation of unrepaired DSBs and, eventually, cell death.

The concept of synthetic lethality with PARP inhibitors extends beyond BRCA1/2-mutated cancers [24]. Other DNA repair deficiencies, such as mutations in other HR pathway genes (e.g., PALB2) or deficiencies in other repair mechanisms such as NER, have also shown sensitivity to PARP inhibitors. These deficiencies create a context in which cancer cells become reliant on PARP-mediated repair pathways, making them susceptible to PARP inhibition.

In breast and ovarian cancer, HRD is associated with a better response to platinum-based chemotherapy and PARP inhibitors. In fact, HRD is a biomarker that is used to predict the response of ovarian cancer to PARP inhibitors [17,25,26,27]. Patients with ovarian cancer who have germline or somatic BRCA1 or BRCA2 mutations, or those patients with high levels of genomic scarring, a hallmark of HRD, have been shown to have better response rates and longer progression-free survival when treated with PARP inhibitors [28,29]. In addition, HRD has been implicated in the response to neoadjuvant chemotherapy, which is provided before surgery to reduce the size of the tumor. Patients with breast cancer who have HRD have been shown to have higher pathologic complete response rates, which is a surrogate endpoint for better survival outcomes [27].

## 7. HRD and Genome-Wide Loss of Heterozygosity (LOH) in Cutaneous Cancers

In cutaneous cancers, HRD can be defined by several molecular and genomic characteristics, including loss of function mutations or epigenetic silencing of key genes involved in the HR pathway, such as BRCA1, BRCA2, RAD51, and PALB2. In addition, HRD can be inferred from the presence of genomic scars, which are genomic rearrangements and copy number alterations that result from impaired DNA repair [18].

**Figure 1:**
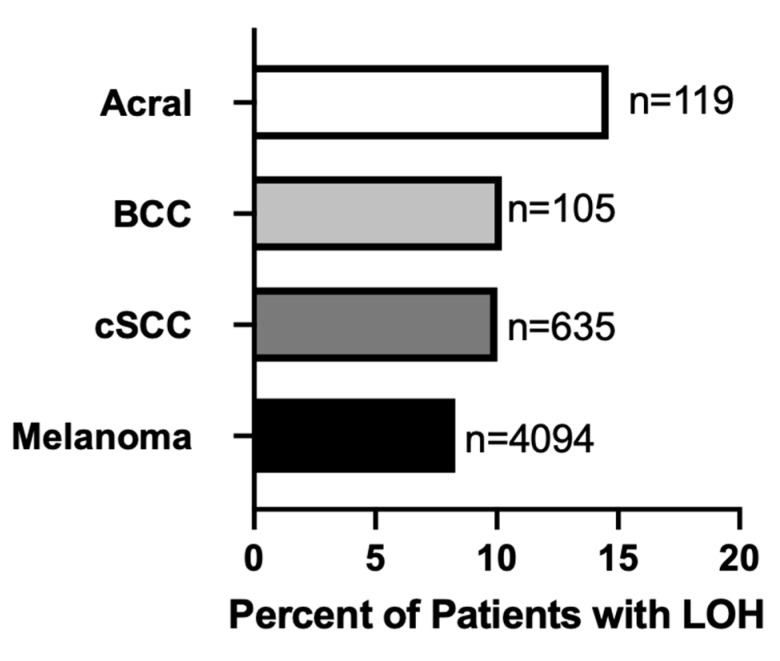
Prevalence of genome-wide LOH-High (≥16% of segments analyzed) among cutaneous cancer (acral, basal cell carcinoma (BCC), cutaneous squamous cell carcinoma (cSCC) and melanoma) patient samples profiled at Caris Life Sciences (n = 4953).

Genome-wide loss of heterozygosity (gLOH) is a marker of HRD in multiple cancers [30]. From a database of over 4500 real-world patient samples submitted to Caris Life Sciences for molecular profiling, we investigated the proportion of cutaneous cancers exhibiting a gLOH-High genomic signature, defined as LOH at ≥16% of segments analyzed. gLOH was detected in 8.29% of all cutaneous melanoma samples (Figure 1). In cutaneous SCC and BCC patients, gLOH-High was detected in 10.03% and 10.2%, respectively (Figure 1). While only 8.57% of cutaneous melanoma were identified as gLOH-High, a notably higher frequency of gLOH-High (14.16%) was observed in acral melanoma samples (Figure 1).

## 8. HRD, PARP Inhibition, and Immunotherapy

Homologous recombination deficiency (HRD) has been implicated in the response to immunotherapy in various cancer types, including breast and ovarian cancer [31]. HRD can influence the tumor microenvironment, immune cell infiltration, and the expression of immune-related genes, which can impact the response to immunotherapy. HRD, particularly in the context of BRCA1/2 mutations, can lead to an increased tumor mutational burden (TMB). TMB is associated with a higher likelihood of neoantigen formation, which can enhance immune recognition and potentially increase the response to immune checkpoint inhibitors [32]. In addition, HRD tumors may exhibit an increased infiltration of immune cells, such as T cells and natural killer (NK) cells [33]. These infiltrating immune cells can contribute to an enhanced antitumor immune response, leading to improved response rates to immunotherapy.

PARP inhibitors have been found to induce immunogenic cell death and promote an immune response [34]. Combining PARP inhibitors with immunotherapy, such as immune checkpoint inhibitors, has shown synergistic effects in preclinical and clinical studies [35]. HRD status has been investigated as a potential predictive biomarker for response to immune checkpoint inhibitors [36].

## 9. HRD Frequency and PARP Inhibition in Melanoma

The incidence of HRD in melanoma patients has been estimated to range from 18–57% in various patient cohorts [37,38,39,40]. The difference in HRD frequency in these studies may be related to the HR–DDR genes that were used for mutation analysis. BRCA1, ARID1A, ATM, ATR, and FANCA, along with concurrent NF1, BRAF, and NRAS mutations, were used in many cases [37,38]. Compared to breast, prostate, and ovarian cancer, there was a higher proportion of melanoma patients with HRD that had BAP1 mutations [38,41]. For a detailed analysis of PARP inhibitor use in melanoma, see [41].

## 10. Clinical Studies

PARP inhibition used as monotherapy has been well-studied among various cancers, including prostate, pancreatic, breast, and ovarian cancers; however, there is limited literature on this relationship in melanoma [42]. Literature on the alterations of HR–DDR mechanisms and the subsequent therapeutic implications has been established in cutaneous melanomas. One study identified that an increased expression of DNA repair genes in melanoma was associated with increased relapse rates and lower chances of responding to chemotherapy [43], highlighting the need for combination therapy in these patients in targeting resistance. A separate study suggested that HR–DDR alterations in cutaneous melanoma are associated with a higher TMB, a known biomarker in predicting response to melanoma immunotherapy [44]. Given the prevalence of HR–DDR in cutaneous melanoma and the promising role of PARP inhibition in targeting HR–DDR melanomas in preclinical models, exploring the clinical efficacy of PARP inhibition in melanoma patients with HR–DDR mutations is strongly warranted.

There have been several clinical trials assessing the effect of combinatory PARP inhibition (with rucaparib or veliparib) with chemotherapy (temozolomide) in treating advanced, chemo-resistant, metastatic melanoma (Table 1) [45,46]. Both studies observed a trend in improvement in progression-free survival (PFS) that ultimately was not statistically significant. Of clinical relevance, both studies observed bone marrow suppression to be a dose-limiting toxicity, and an 80% dose reduction was necessary for safe delivery of the combinatorial treatments. While the trend towards significance implies that future studies with larger sample sizes may unearth this relationship, the authors in these highlighted studies did not categorize patients based on their HR–DDR statuses, an effect modification that may very well have led to statistical significance. For example, a case report evaluating olaparib monotherapy in treating a patient with HR–DDR metastatic melanoma (PALB2 mutation) identified a partial response, despite initial progression on immunotherapy (ipilimumab and nivolumab) [47], highlighting the importance of considering HR–DDR status in optimizing melanoma management.

Among patients with melanoma, 40–60% develop acquired or de novo resistance to immunotherapy [54,55], thus necessitating newer treatment modalities such as combinatory PARP inhibition and immunotherapy. In 2021, we published a case report describing a near-complete response in a patient with HRD metastatic melanoma (germline CHEK2 mutation and somatic BRCA2, TP53, NF1, and ATRX mutations) undergoing combinatory treatment with olaparib and nivolumab (after initial progression on nivolumab maintenance monotherapy), with clearance of all somatic mutations following treatment [52]. Similarly, another HRD patient (LOH 32.9%) with metastatic melanoma showed a near-complete response when treated with combinatory olaparib and nivolumab [51]. This patient progressed on initial nivolumab monotherapy and mutational clearance was observed after combinatorial therapy [51]. Unlike in our previous case report, this patient had LOH 32.9%, despite not having any HR–DDR gene mutations, suggesting that other DDR mutations can contribute to “HRDness” and still benefit from PARP inhibition therapy, further highlighting the importance in designating HRD status in melanoma patients.

In a recently published case series, we documented the response of three additional melanoma patients with HRD–LOH score ranging from 28–58% to PARP inhibitor therapy after initial failure of immune checkpoint therapy, with a durable response observed in one patient (Zhou JNCCN 2023). Several studies have also identified utility in combinatory PARP inhibition (niraparib or talazoparib) and radiotherapy, via the induction of G2/M arrest and subsequent cell death, suggesting that PARP inhibition can sensitize melanoma to varying therapeutic modalities [49,56].

## 11. PARP Inhibition in Non-Melanoma Skin Cancer (NMSC)

Unlike melanoma, there are few studies assessing the prevalence of HRD alterations in NMSCs. Zhang et al.’s 2022 pan-cancer analysis of 45,604 patients with diverse tumors identified non-melanoma skin cancers having the highest frequency of PARP1 mutations among cancer types (8.98%, 44/490) [57]. Advanced stages of NMSCs are more prone to PARP alterations when compared to early-stage disease, which raises concern for possible de novo PARP inhibitor resistance [57].

## 12. Cutaneous Squamous Cell Carcinoma

A few studies have documented the involvement of PARP in cSCC while investigating other therapies [58,59]. A large study of 1873 patients reported an increased risk of invasive cutaneous SCC among men with a family history of BRCA1 mutations [60]. Despite having a wide confidence interval (6.02; 1.96–14.05), this relationship between cSCC risk and BRCA1 mutations is clinically interesting. In addition, previous studies have implicated BRCA1-alterations in the development of cSCC [61,62]. Clinical studies on PARP inhibition in cSCC are lacking. A recent case report assessed the efficacy of fluzoparib in the treatment of immunotherapy refractory, BRCA2 positive, PD-L1 negative metastatic cSCC (Table 1). The authors observed a progression-free survival of five months, tumor stability, and regression of lung metastasis after fluzoparib monotherapy [50]. These promising results highlight the importance in subtyping SCC in guiding management—BRCA-altered SCC can display sensitivity to PARP inhibition despite resistance to immunotherapy.

## 13. Cutaneous Basal Cell Carcinoma

While p53 mutations (commonly via UV and ionizing radiation) are common in BCC, genetic alterations have been shown to underlie BCC pathogenesis, namely the hedgehog (Hh) pathway. The alteration of the Hh pathway, which typically plays a role in embryonic development [63,64], has been shown to contribute to BCC development, particularly through mutations in the PTCH1 gene, an integral part of the Hh signaling pathway [65]. Interestingly, PARP1 has been shown to interact with PTCH1 in the pathogenesis of BCC, particularly in response to environmental stressors such as radiation. Tanori et al. observed Ptch+/− mice with PARP1 deletion to be highly prone to developing radiation-induced BCCs [66]. However, studies involving other DDR-associated genes, such as XRCC1-3, whose protein products have been shown to interact with PARP1, observed that alterations conferred a decreased risk for BCC [66,67,68]. These contradictory results highlight a gap in knowledge on PARP inhibition and BCC development and treatment, thus warranting more studies to identify specific interactions between various DDR genes and PARP in BCC pathogenesis. Currently, no clinical studies were identified assessing PARP inhibition in cutaneous BCC; however, more preclinical data are warranted before attempting a transition to clinical studies.

## 14. Merkel Cell Carcinoma

Merkel cell carcinoma (MCC) is a rare and aggressive neuroendocrine skin cancer whose exact cell of origin and pathogenesis are unknown; however, its development is associated with Merkel cell polyomavirus (MCPyV) and UV radiation [69,70]. As such, MCC can be subdivided based on pathogenesis into MCPyV-positive and MCPyV-negative where MCPyV-negative MCC have a high number of UV-associated DNA mutations, particularly in tumor suppressor genes (RB1, TP53), which lead to uncontrolled cell division [69]. It has been noted that MCPyV-negative MCC shares a similar mutational profile to that of small cell lung cancer (SCLC) [70], which demonstrates a high expression of DNA repair proteins, particularly PARP1 [71], thus prompting investigation by Ferrarotto in 2018 into the use of PARP1 as a therapeutic target for MCC. This small retrospective study found that 74% (N = 19) of MCC samples expressed high levels of PARP1, 64% were positive for mutations in DNA damage repair genes (indicative of MCPyV-negative MCC), and sensitivity to olaparib was seen in the Merkel cell carcinoma line with highest PARP1 expression [70]. These clinical findings should prompt clinical trials into the use of PARP inhibitors as monotherapy or combination therapy for MCC, especially the MCPyV-negative subtype.

## 15. Adverse Effects of PARP Inhibitors

PARP inhibitors use synthetic lethality to specifically target malignant cells by effectively binding to PARP enzyme. This interaction inhibits the process of PARylation and causes PARP to trap in cancer cells, leading to cell death in DNA repair-deficient cells [72]. PARP proteins are a family of proteins that each have similar yet different functions; thus, the adverse effects vary based on the affinity of a PARP inhibitor for a particular PARP protein [73]. PARP inhibitors can also be differentiated by their PARP-trapping abilities, with greater PARP trapping being associated with high myelosuppression [73]. Therefore, each PARP inhibitor should be treated as a unique entity.

The adverse effects of PARP inhibitors have not been well studied in cutaneous cancers, but the adverse effects reported in other types of malignancies have yielded similar results. A meta-analysis compared the safety and tolerability of approved PARP inhibitors (fluzoparib, olaparib, rucaparib, niraparib, or talazoparib) in 10 head-to-head phase II and phase III randomized controlled trials (RCTs), with either placebo or chemotherapy, in cancer patients [72]. The four PARP inhibitors were comparable in terms of serious adverse events (SAE) and adverse events (AE) leading to discontinuation of treatment [72]. This suggests that there is a similar toxicity profile; however, statistically significant differences were seen in the interruption of treatment and dose reduction due to AE, with the highest risk seen in talazoparib and the lowest risk seen in niraparib [72]. Exploring and comparing adverse events between PARP inhibitors (niraparib, olaparib, and rucaparib) found that hematologic toxicity was common across the PARP-inhibitors drug class, with anemia being the most common and likely associated with activity against PARP 2. Compared to olaparib and rucaparib, niraparib was associated with more hematologic toxicities with more severe and increased incidence of neutropenia and thrombocytopenia [73]. Gastrointestinal (GI) toxicities, especially nausea, have also been noted in all PARP inhibitors; these are treated similarly to those of chemotherapy-induced GI toxicities. PARP inhibitors are associated with an increase in creatinine concentration (except for niraparib), but this may not reflect a true decline in renal function, so GFR should be monitored to avoid inappropriate dose reductions or discontinuation [73]. As rucaparib carries the greatest risk of increasing creatinine, its use should be avoided in patients with pre-existing kidney disease [74]. Despite niraparib’s lack of renal toxicity, it is associated with cardiovascular toxicity (hypertension and myocardial dysfunction). As such, blood pressure and heart rate should be monitored closely, especially in patients with cardiovascular disease [73,74]. The development of secondary malignancies such as myelodysplastic syndrome and acute myeloid leukemia have been reported. However, these are rare complications, and it is unclear whether they are directly related to PARP inhibitors. Furthermore, some patients had prior platinum-based chemotherapy/DNA-damaging therapies or a history of bone marrow dysplasia/other primary cancer [73]. The highlighted differences between PARP inhibitors should be taken into consideration in order to individualize and optimize treatment for patients.

Despite the above-mentioned adverse effects, the use of PARP inhibitors has been shown to improve patients’ overall quality of life (QOL). QOL studies have attributed this to a decrease in disease-specific symptoms following disease regression, drug tolerability while on maintenance PARP therapy, and delayed deterioration, compared to chemotherapy [73,75]. Even though PARP inhibitors are not without their adverse effects, their adverse effects are similar to those of other currently available therapies and are manageable with adjunctive therapies, but they have the benefit of improved QOL. Therefore, PARP-inhibitor therapy in cutaneous cancers is likely to be well-tolerated.

## 16. Resistance to PARP Inhibitors

The use of PARP inhibitors has not been without challenges, due to the development of resistance in HRD tumor cells. While not explored in cutaneous malignancies, it has been a point of clinical concern in other HRD-deficient tumors [76]. Interestingly, resistance to platinum-based chemotherapies is a strong predictor for PARP-inhibitor resistance [76]; thus, this could be used to predict the likelihood of PARP-inhibitor resistance in this specific subset of cancers. Resistance develops via four main mechanisms: (1) drug availability, (2) ability to affect (de)PARylation enzymes, (3) restoration of homologous recombination (HR), or (4) restoration of replication fork stability [76,77,78,79]. Targeted approaches to combat tumors with PARP-inhibitor resistance are limited, but the data seem promising. Combination therapies with PARP inhibitors aimed at targeting alternative HR pathways, such as the 53BP1–RIF1–REV7–shielding end-protection pathway, have led to the development of RAD 52 inhibitors [77]. RAD 52 is a small DNA repair protein whose depletion has been associated with a different type of synthetic lethality in HRD tumor cells, compared to PARP inhibitors; this area requires further study in human models [80]. The indirect inhibition of HR with the use of therapies targeting EGFR, IGF1R, VEGF, or the PI3K–AKT pathway in conjunction with PARP inhibitors seems to be another viable strategy [77]. In a cohort study looking at the use of bevacizumab (anti-VEGF) with olaparib or niraparib in patients with ovarian cancer, there was an increase in median progression-free survival in two cohorts, even those with HR-proficient tumors [48,53]. This combination therapy results in the additive effects of cell-cycle progression disruption and was found to be superior to placebo and monotherapy [48,53,77]. PARP-inhibitor resistance has been associated with an increase in ATR-CHK1 pathway activity, due to PARP1 trapping, which results in the phosphorylation of several proteins that aid replication fork stability and facilitates the progression of DNA synthesis in PARP-inhibitor resistant tumor cells by overriding cell-cycle checkpoint signaling [77,79,81], thus increasing the sensitivity to ATR inhibitors [82]. It was also demonstrated that a PARP-inhibitor–ATR-inhibitor combination was superior to ATR-inhibitor monotherapy in PARP-inhibitor resistant cells [82]. Compared to placebo in the preclinical setting, ATR inhibitor showed a five-fold increase in apoptosis of PARP inhibitor/platinum resistant BRCA mutated cells (*p* <  0.001), while the PARP-inhibitor–ATR-inhibitor combination led to 1.8-fold and 1.6-fold increases (*p* < 0.001) in apoptosis vs. the ATR inhibitor [82]. The currently ongoing phase 2 clinical trials, OPALCO and CAPRI, are considering the combination of olaparib with the ATR inhibitor, AZD6738, in metastatic solid tumors and recurrent ovarian tumors, respectively [81]. Another potential approach is the combination of PARP inhibitors with immune-checkpoint inhibitors, such as anti-PD-1 antibodies in BRCA-deficient tumors [77]. PARP inhibitors have been shown to induce the expression of PDL-1 and to enhance the antitumor effects of anti-PD-1 antibodies in mouse models of breast and ovarian cancer; only a small cohort of patients have been studied with this combination; thus, this requires further investigation [77]. DNA polymerase θ (POLQ) inhibitors have become an area of considerable research interest in the field of PARP-inhibitor resistance [77]. BRCA-deficient cells are able to upregulate microhomology-mediated end-joining (MMEJ) as a compensatory mechanism to facilitate DSB DNA repair in the absence of HR [77]. MMEJ is driven by POLQ, which joins two broken DNA strands based on short regions of sequence homology, and this creates a detectable MMEJ-characteristic pattern of mutations in BRCA1/2-deficient tumors [77]. POLQ has not only demonstrated efficacy in tumors with acquired PARP-inhibitor resistance, but also in preventing the emergence of PARP-inhibitor resistance in PARP-inhibitor-naive HR deficient cells [77]. These inhibitors have a synthetic lethality mechanism, and they also suppress the genomic instability that arises from HRD, thereby promoting the emergence of HR-deficient cancers of a “mutator phenotype” [77,83]. As PARP inhibitor therapy is now being considered as a treatment modality for cancers that have failed current lines of therapy, there is an urgency for further insight into preventing and overcoming PARP-inhibitor resistance.

In conclusion, we have explored recent findings on HRD and PARP inhibitors in cutaneous malignancies and highlighted parallels from breast and ovarian cancer. In addition, we have discussed the therapy complications from PARP inhibitor. Additional randomized controlled studies using PARP inhibitor in patients with HRD as single or combined therapy may improve therapeutic options in cutaneous malignancies.

## Figures and Tables

**Table 1:** PARP Inhibitor Therapies.

PARP Inhibitor	PARP Target	Other Therapy (if Applicable)	Clinical Study	HRD Cancer Types
Rucaparib	PARP1, 2 & 3	Temozolamide	[46]	metastatic melanoma
Veliparib	PARP1 & 2	Temozolamide	[45]	metastatic melanoma
Niraparib	PARP1 & 2	Bevacizumab	[48]	platinum-sensitive epithelial ovarian cancer
		Radiotherapy	[49]	melanoma
Talazoparib	PARP1 & 2	Radiotherapy	[49]	melanoma
Fluzoparib	PARP1	n/a	[50]	chemo refractory metastatic cSCC
Olaparib	PARP1 & 2	n/a	[27,29]	BRCA mutated breast cancer
		n/a	[28]	advanced ovarian cancer
		n/a	[47]	metastatic melanoma
		Nivolumab	[51,52]	relapsed metastatic melanoma
		Bevacizumab	[53]	ovarian cancer

n/a means not applicable.

## Data Availability

Data sharing is not applicable to this article.

## References

[1] Guy G.P., Thomas C.C., Thompson T., Watson M., Massetti G.M., Richardson L.C. (2015). Vital signs: Melanoma incidence and mortality trends and projections—United States, 1982–2030. MMWR Morb. Mortal. Wkly. Rep..

[2] Siegel R.L., Miller K.D., Fuchs H.E., Jemal A. (2022). Cancer statistics, 2022. CA Cancer J. Clin..

[3] Que S.K.T., Zwald F.O., Schmults C.D. (2018). Cutaneous squamous cell carcinoma: Incidence, risk factors, diagnosis, and staging. J. Am. Acad. Derm..

[4] Arnold M., Singh D., Laversanne M., Vignat J., Vaccarella S., Meheus F., Cust A.E., de Vries E., Whiteman D.C., Bray F. (2022). Global Burden of Cutaneous Melanoma in 2020 and Projections to 2040. JAMA Derm..

[5] Cameron M.C., Lee E., Hibler B.P., Barker C.A., Mori S., Cordova M., Nehal K.S., Rossi A.M. (2019). Basal cell carcinoma: Epidemiology; pathophysiology; clinical and histological subtypes; and disease associations. J. Am. Acad. Derm..

[6] Srinivas U.S., Tan B.W.Q., Vellayappan B.A., Jeyasekharan A.D. (2019). ROS and the DNA damage response in cancer. Redox Biol..

[7] Spivak G. (2015). Nucleotide excision repair in humans. DNA Repair.

[8] Rastogi R.P., Richa K.A., Tyagi M.B., Sinha R.P. (2010). Molecular mechanisms of ultraviolet radiation-induced DNA damage and repair. J. Nucleic Acids.

[9] Brambullo T., Colonna M.R., Vindigni V., Piaserico S., Masciopinto G., Galeano M., Costa A.L., Bassetto F. (2022). Xeroderma Pigmentosum: A Genetic Condition Skin Cancer Correlated-A Systematic Review. Biomed. Res. Int..

[10] Krokan H.E., Bjørås M. (2013). Base excision repair. Cold Spring Harb. Perspect. Biol..

[11] Li Z., Pearlman A.H., Hsieh P. (2016). DNA mismatch repair and the DNA damage response. DNA Repair.

[12] Lieber M.R. (2010). The mechanism of double-strand DNA break repair by the nonhomologous DNA end-joining pathway. Annu. Rev. Biochem..

[13] Pannunzio N.R., Watanabe G., Lieber M.R. (2018). Nonhomologous DNA end-joining for repair of DNA double-strand breaks. J. Biol. Chem..

[14] Li X., Heyer W.-D. (2008). Homologous recombination in DNA repair and DNA damage tolerance. Cell Res..

[15] Murai J., Huang S.Y., Das B.B., Renaud A., Zhang Y., Doroshow J.H., Ji J., Takeda S., Pommier Y. (2012). Trapping of PARP1 and PARP2 by Clinical PARP Inhibitors. Cancer Res..

[16] Ali A.A.E., Timinszky G., Arribas-Bosacoma R., Kozlowski M., Hassa P.O., Hassler M., Ladurner A.G., Pearl L.H., Oliver A.W. (2012). The zinc-finger domains of PARP1 cooperate to recognize DNA strand breaks. Nat. Struct. Mol. Biol..

[17] Kohn E.C., Lee J.M., Ivy S.P. (2017). The HRD Decision-Which PARP Inhibitor to Use for Whom and When. Clin. Cancer Res..

[18] Watkins J.A., Irshad S., Grigoriadis A., Tutt A.N. (2014). Genomic scars as biomarkers of homologous recombination deficiency and drug response in breast and ovarian cancers. Breast Cancer Res..

[19] Cruz C., Castroviejo-Bermejo M., Gutiérrez-Enríquez S., Llop-Guevara A., Ibrahim Y.H., Gris-Oliver A., Bonache S., Morancho B., Bruna A., Rueda O.M. (2018). RAD51 foci as a functional biomarker of homologous recombination repair and PARP inhibitor resistance in germline BRCA-mutated breast cancer. Ann. Oncol..

[20] Nakanishi K., Cavallo F., Brunet E., Jasin M. (2011). Homologous recombination assay for interstrand cross-link repair. Methods Mol. Biol..

[21] Pierce A.J., Jasin M. (2005). Measuring recombination proficiency in mouse embryonic stem cells. Methods Mol. Biol..

[22] Stults D.M., Killen M.W., Pierce A.J. (2014). The sister chromatid exchange (SCE) assay. Methods Mol. Biol..

[23] Rouleau M., Patel A., Hendzel M.J., Kaufmann S.H., Poirier G.G. (2010). PARP inhibition: PARP1 and beyond. Nat. Rev. Cancer.

[24] Livraghi L., Garber J.E. (2015). PARP inhibitors in the management of breast cancer: Current data and future prospects. BMC Med..

[25] Mirza M.R., Monk B.J., Herrstedt J., Oza A.M., Mahner S., Redondo A., Fabbro M., Ledermann J.A., Lorusso D., Vergote I. (2016). Niraparib Maintenance Therapy in Platinum-Sensitive, Recurrent Ovarian Cancer. N. Engl. J. Med..

[26] Konstantinopoulos P.A., Luo W., Liu J.F., Gulhan D.C., Krasner C., Ishizuka J.J., Gockley A.A., Buss M., Growdon W.B., Crowe H. (2019). Phase II Study of Avelumab in Patients With Mismatch Repair Deficient and Mismatch Repair Proficient Recurrent/Persistent Endometrial Cancer. J. Clin. Oncol..

[27] Tutt A.N.J., Garber J.E., Kaufman B., Viale G., Fumagalli D., Rastogi P., Gelber R.D., de Azambuja E., Fielding A., Balmaña J. (2021). Adjuvant Olaparib for Patients with BRCA1- or BRCA2-Mutated Breast Cancer. N. Engl. J. Med..

[28] Banerjee S., Moore K.N., Colombo N., Scambia G., Kim B.G., Oaknin A., Friedlander M., Lisyanskaya A., Floquet A., Leary A. (2021). Maintenance olaparib for patients with newly diagnosed advanced ovarian cancer and a BRCA mutation (SOLO1/GOG 3004): 5-year follow-up of a randomised, double-blind, placebo-controlled, phase 3 trial. Lancet Oncol..

[29] Robson M., Im S.A., Senkus E., Xu B., Domchek S.M., Masuda N., Delaloge S., Li W., Tung N., Armstrong A. (2017). Olaparib for Metastatic Breast Cancer in Patients with a Germline BRCA Mutation. N. Engl. J. Med..

[30] Wagener-Ryczek S., Merkelbach-Bruse S., Siemanowski J. (2021). Biomarkers for Homologous Recombination Deficiency in Cancer. J. Pers. Med..

[31] Milanesio M.C., Giordano S., Valabrega G. (2020). Clinical Implications of DNA Repair Defects in High-Grade Serous Ovarian Carcinomas. Cancers.

[32] Klempner S.J., Fabrizio D., Bane S., Reinhart M., Peoples T., Ali S.M., Sokol E.S., Frampton G., Schrock A.B., Anhorn R. (2020). Tumor Mutational Burden as a Predictive Biomarker for Response to Immune Checkpoint Inhibitors: A Review of Current Evidence. Oncologist.

[33] Morse C.B., Toukatly M.N., Kilgore M.R., Agnew K.J., Bernards S.S., Norquist B.M., Pennington K.P., Garcia R.L., Liao J.B., Swisher E.M. (2019). Tumor infiltrating lymphocytes and homologous recombination deficiency are independently associated with improved survival in ovarian carcinoma. Gynecol. Oncol..

[34] Lee E.K., Konstantinopoulos P.A. (2020). PARP inhibition and immune modulation: Scientific rationale and perspectives for the treatment of gynecologic cancers. Adv. Med. Oncol..

[35] Vikas P., Borcherding N., Chennamadhavuni A., Garje R. (2020). Therapeutic Potential of Combining PARP Inhibitor and Immunotherapy in Solid Tumors. Front. Oncol..

[36] Zhou Z., Ding Z., Yuan J., Shen S., Jian H., Tan Q., Yang Y., Chen Z., Luo Q., Cheng X. (2022). Homologous recombination deficiency (HRD) can predict the therapeutic outcomes of immuno-neoadjuvant therapy in NSCLC patients. J. Hematol. Oncol..

[37] Kim K.B., Soroceanu L., de Semir D., Millis S.Z., Ross J., Vosoughi E., Dar A.A., Nosrati M., Desprez P.Y., Ice R. (2021). Prevalence of Homologous Recombination Pathway Gene Mutations in Melanoma: Rationale for a New Targeted Therapeutic Approach. J. Investig. Derm..

[38] Heeke A.L., Pishvaian M.J., Lynce F., Xiu J., Brody J.R., Chen W.J., Baker T.M., Marshall J.L., Isaacs C. (2018). Prevalence of Homologous Recombination-Related Gene Mutations Across Multiple Cancer Types. JCO Precis. Oncol..

[39] Liu H., Zhang Y., Ding F., Zhang Y., Liang X., Lou F., Cao S., Wang H. (2021). Frequency of homologous recombination deficiency gene mutations in melanoma and its relevance to the immunotherapeutic response. J. Clin. Oncol..

[40] Kim H., Ahn S., Kim H., Hong J.Y., Lee J., Park S.H., Park J.O., Park Y.S., Lim H.Y., Kang W.K. (2022). The prevalence of homologous recombination deficiency (HRD) in various solid tumors and the role of HRD as a single biomarker to immune checkpoint inhibitors. J. Cancer Res. Clin. Oncol..

[41] Chan W.Y., Brown L.J., Reid L., Joshua A.M. (2021). PARP Inhibitors in Melanoma-An Expanding Therapeutic Option?. Cancers.

[42] Cong K., Peng M., Kousholt A.N., Lee W.T.C., Lee S., Nayak S., Krais J., VanderVere-Carozza P.S., Pawelczak K.S., Calvo J. (2021). Replication gaps are a key determinant of PARP inhibitor synthetic lethality with BRCA deficiency. Mol. Cell.

[43] Jewell R., Conway C., Mitra A., Randerson-Moor J., Lobo S., Nsengimana J., Harland M., Marples M., Edward S., Cook M. (2010). Patterns of expression of DNA repair genes and relapse from melanoma. Clin. Cancer Res..

[44] Chiu T.Y., Lin R.W., Huang C.J., Yeh D.W., Wang Y.C. (2021). DNA Damage Repair Gene Set as a Potential Biomarker for Stratifying Patients with High Tumor Mutational Burden. Biology.

[45] Middleton M.R., Friedlander P., Hamid O., Daud A., Plummer R., Falotico N., Chyla B., Jiang F., McKeegan E., Mostafa N.M. (2015). Randomized phase II study evaluating veliparib (ABT-888) with temozolomide in patients with metastatic melanoma. Ann. Oncol..

[46] Plummer R., Lorigan P., Steven N., Scott L., Middleton M.R., Wilson R.H., Mulligan E., Curtin N., Wang D., Dewji R. (2013). A phase II study of the potent PARP inhibitor, Rucaparib (PF-01367338, AG014699), with temozolomide in patients with metastatic melanoma demonstrating evidence of chemopotentiation. Cancer Chemother. Pharm..

[47] Lau B., Menzies A.M., Joshua A.M. (2021). Ongoing partial response at 6 months to olaparib for metastatic melanoma with somatic PALB2 mutation after failure of immunotherapy: A case report. Ann. Oncol..

[48] Mirza M.R., Bergmann T.K., Mau-Sørensen M., Christensen R.D., Åvall-Lundqvist E., Birrer M.J., Jørgensen M., Roed H., Malander S., Nielsen F. (2019). A phase I study of the PARP inhibitor niraparib in combination with bevacizumab in platinum-sensitive epithelial ovarian cancer: NSGO AVANOVA1/ENGOT-OV24. Cancer Chemother. Pharm..

[49] Jonuscheit S., Jost T., Gajdošová F., Wrobel M., Hecht M., Fietkau R., Distel L. (2021). PARP Inhibitors Talazoparib and Niraparib Sensitize Melanoma Cells to Ionizing Radiation. Genes.

[50] Sun X., Chen W., Qu X., Chen Y. (2022). Case Report: Fluzoparib for multiple lines of chemotherapy refractory in metastatic cutaneous squamous cell carcinoma with BRCA2 pathogenic mutation. Front. Pharm..

[51] Khaddour K., Ansstas M., Ansstas G. (2021). Clinical outcomes and longitudinal circulating tumor DNA changes after treatment with nivolumab and olaparib in immunotherapy relapsed melanoma with detected homologous recombination deficiency. Cold Spring Harb. Mol. Case Stud..

[52] Khaddour K., Ansstas M., Visconti J., Ansstas G. (2021). Mutation clearance and complete radiologic resolution of immunotherapy relapsed metastatic melanoma after treatment with nivolumab and olaparib in a patient with homologous recombinant deficiency: Any role for PARP inhibitors and checkpoint blockade?. Ann. Oncol..

[53] Ray-Coquard I., Pautier P., Pignata S., Pérol D., González-Martín A., Berger R., Fujiwara K., Vergote I., Colombo N., Mäenpää J. (2019). Olaparib plus Bevacizumab as First-Line Maintenance in Ovarian Cancer. N. Engl. J. Med..

[54] Wolchok J.D., Chiarion-Sileni V., Gonzalez R., Rutkowski P., Grob J.J., Cowey C.L., Lao C.D., Wagstaff J., Schadendorf D., Ferrucci P.F. (2017). Overall Survival with Combined Nivolumab and Ipilimumab in Advanced Melanoma. N. Engl. J. Med..

[55] Larkin J., Chiarion-Sileni V., Gonzalez R., Grob J.J., Rutkowski P., Lao C.D., Cowey C.L., Schadendorf D., Wagstaff J., Dummer R. (2019). Five-Year Survival with Combined Nivolumab and Ipilimumab in Advanced Melanoma. N. Engl. J. Med..

[56] Weigert V., Jost T., Hecht M., Knippertz I., Heinzerling L., Fietkau R., Distel L.V. (2020). PARP inhibitors combined with ionizing radiation induce different effects in melanoma cells and healthy fibroblasts. BMC Cancer.

[57] Zhang X., Wang Y., Gari A, Qu C., Chen J. (2021). Pan-Cancer Analysis of PARP1 Alterations as Biomarkers in the Prediction of Immunotherapeutic Effects and the Association of Its Expression Levels and Immunotherapy Signatures. Front. Immunol..

[58] Zhang Q.L., Lian D.D., Zhu M.J., Li X.M., Lee J.K., Yoon T.J., Lee J.H., Jiang R.H., Kim C.D. (2019). Antitumor Effect of Albendazole on Cutaneous Squamous Cell Carcinoma (SCC) Cells. Biomed. Res. Int..

[59] Farkas B., Magyarlaki M., Csete B., Nemeth J., Rabloczky G., Bernath S., Literáti Nagy P., Sümegi B. (2002). Reduction of acute photodamage in skin by topical application of a novel PARP inhibitor. Biochem. Pharm..

[60] Johannsson O., Loman N., Möller T., Kristoffersson U., Borg A., Olsson H. (1999). Incidence of malignant tumours in relatives of BRCA1 and BRCA2 germline mutation carriers. Eur. J. Cancer.

[61] Ginsburg O.M., Kim-Sing C., Foulkes W.D., Ghadirian P., Lynch H.T., Sun P., Narod S.A. (2010). BRCA1 and BRCA2 families and the risk of skin cancer. Fam. Cancer.

[62] Navaraj A., Mori T., El-Deiry W.S. (2005). Cooperation between BRCA1 and p53 in repair of cyclobutane pyrimidine dimers. Cancer Biol..

[63] Wicking C., Smyth I., Bale A. (1999). The hedgehog signalling pathway in tumorigenesis and development. Oncogene.

[64] Ingham P.W., Placzek M. (2006). Orchestrating ontogenesis: Variations on a theme by sonic hedgehog. Nat. Rev. Genet..

[65] Acocella A., Sacco R., Bertolai R., Sacco N. (2009). Genetic and clinicopathologic aspects of Gorlin-Goltz syndrome (NBCCS): Presentation of two case reports and literature review. Minerva Stomatol..

[66] Tanori M., Mancuso M., Pasquali E., Leonardi S., Rebessi S., Di Majo V., Guilly M.N., Giangaspero F., Covelli V., Pazzaglia S. (2008). PARP-1 cooperates with Ptc1 to suppress medulloblastoma and basal cell carcinoma. Carcinogenesis.

[67] Schultz N., Lopez E., Saleh-Gohari N., Helleday T. (2003). Poly(ADP-ribose) polymerase (PARP-1) has a controlling role in homologous recombination. Nucleic Acids Res..

[68] Lindahl T., Satoh M.S., Poirier G.G., Klungland A. (1995). Post-translational modification of poly(ADP-ribose) polymerase induced by DNA strand breaks. Trends Biochem. Sci..

[69] Naseri S., Steiniche T., Ladekarl M., Langer L.R., Tabaksblat E., Junker N., Chakera A.H. (2021). Merkel cell carcinoma. Ugeskr Laeger.

[70] Ferrarotto R., Cardnell R., Su S., Diao L., Eterovic A.K., Prieto V., Morrisson W.H., Wang J., Kies M.S., Glisson B.S. (2018). Poly ADP-ribose polymerase-1 as a potential therapeutic target in Merkel cell carcinoma. Head Neck.

[71] Byers L.A., Wang J., Nilsson M.B., Fujimoto J., Saintigny P., Yordy J., Giri U., Peyton M., Fan Y.H., Diao L. (2012). Proteomic profiling identifies dysregulated pathways in small cell lung cancer and novel therapeutic targets including PARP1. Cancer Discov..

[72] Cai Z., Liu C., Chang C., Shen C., Yin Y., Yin X., Jiang Z., Zhao Z., Mu M., Cao D. (2021). Comparative safety and tolerability of approved PARP inhibitors in cancer: A systematic review and network meta-analysis. Pharm. Res..

[73] LaFargue C.J., Dal Molin G.Z., Sood A.K., Coleman R.L. (2019). Exploring and comparing adverse events between PARP inhibitors. Lancet Oncol..

[74] Hennes E.R., Dow-Hillgartner E.N., Bergsbaken J.J., Piccolo J.K. (2020). PARP-inhibitor potpourri: A comparative review of class safety, efficacy, and cost. J. Oncol. Pharm. Pract..

[75] Barchiesi G., Roberto M., Verrico M., Vici P., Tomao S., Tomao F. (2021). Emerging Role of PARP Inhibitors in Metastatic Triple Negative Breast Cancer. Current Scenario and Future Perspectives. Front. Oncol..

[76] Noordermeer S.M., van Attikum H. (2019). PARP Inhibitor Resistance: A Tug-of-War in BRCA-Mutated Cells. Trends Cell. Biol..

[77] Dias M.P., Moser S.C., Ganesan S., Jonkers J. (2021). Understanding and overcoming resistance to PARP inhibitors in cancer therapy. Nat. Rev. Clin. Oncol..

[78] Li H., Liu Z.Y., Wu N., Chen Y.C., Cheng Q., Wang J. (2020). PARP inhibitor resistance: The underlying mechanisms and clinical implications. Mol. Cancer.

[79] D’Andrea A.D. (2018). Mechanisms of PARP inhibitor sensitivity and resistance. DNA Repair.

[80] Hengel S.R., Spies M.A., Spies M. (2017). Small-Molecule Inhibitors Targeting DNA Repair and DNA Repair Deficiency in Research and Cancer Therapy. Cell Chem. Biol..

[81] Serra V., Wang A.T., Castroviejo-Bermejo M., Polanska U.M., Palafox M., Herencia-Ropero A., Jones G.N., Lai Z., Armenia J., Michopoulos F. (2022). Identification of a Molecularly-Defined Subset of Breast and Ovarian Cancer Models that Respond to WEE1 or ATR Inhibition, Overcoming PARP Inhibitor Resistance. Clin. Cancer Res..

[82] Kim H., Xu H., George E., Hallberg D., Kumar S., Jagannathan V., Medvedev S., Kinose Y., Devins K., Verma P. (2020). Combining PARP with ATR inhibition overcomes PARP inhibitor and platinum resistance in ovarian cancer models. Nat. Commun..

[83] Zatreanu D., Robinson H.M.R., Alkhatib O., Boursier M., Finch H., Geo L., Grande D., Grinkevich V., Heald R.A., Langdon S. (2021). Polθ inhibitors elicit BRCA-gene synthetic lethality and target PARP inhibitor resistance. Nat. Commun..

